# An Integrated Analysis of Factors Influencing Acceptance of Care Robots Among Older Korean Adults

**DOI:** 10.3390/healthcare14030322

**Published:** 2026-01-27

**Authors:** Hee Jeong Yoon, Si Woo Ban, Yeo Min Han, Hye Ri Shin, Young Sun Kim, Won Chul Shin, Seung Don Yoo, Ji Ho Park

**Affiliations:** 1 Department of Gerontology, AgeTech-Service Convergence Major, Kyung Hee University, Yongin-si 17104, Gyeonggi-do, Republic of Korea; yoonhj@khu.ac.kr (H.J.Y.); bansiwoo@khu.ac.kr (S.W.B.); 2024510036@khu.ac.kr (Y.M.H.); zisoa@khu.ac.kr (H.R.S.); jihopark@khu.ac.kr (J.H.P.); 2 Department of Medicine, AgeTech-Service Convergence Major, Kyung Hee University, Seoul 02447, Republic of Korea; shinwc@khu.ac.kr; 3 Department of Rehabilitation Medicine, Kyung Hee University College of Medicine, Kyung Hee University Hospital at Gangdong, Seoul 05278, Republic of Korea; kidlife@khu.ac.kr

**Keywords:** care robots, self-efficacy, technology enthusiasm, technology acceptance, AgeTech

## Abstract

**Background**: As populations rapidly age, care robots have been proposed as a promising solution, supporting independent living and alleviating care burdens. However, acceptance of care robots among older adults remains limited. This study examined the relative contributions of demographic, health-related, digital competence, and technology-related psychological factors to care robot acceptance among older adults in South Korea. **Methods**: A cross-sectional survey was conducted with 506 community-dwelling older adults. Hierarchical multiple regression analyses were used to identify predictors of care robot acceptance, sequentially entering demographic characteristics, health-related factors, digital competence, and technology-related psychological variables. **Results**: Demographic and health-related factors were initially associated with care robot acceptance, but their effects diminished after accounting for digital competence and psychological variables. In the final model, technology-related psychological factors—particularly technology use self-efficacy and technology enthusiasm—were the strongest predictors of acceptance, while most demographic and health variables became non-significant, with the exception of instrumental activities of daily living (IADLs). **Conclusions**: These findings indicate that psychological readiness and confidence in technology use outweigh demographic or health characteristics in shaping older adults’ acceptance of care robots. Interventions and design strategies that enhance self-efficacy, foster positive engagement, and support functional independence may be critical for promoting the effective and sustainable adoption of care robots in aging societies.

## 1. Introduction

With the global population aging, the number of older adults with complex health needs is increasing, leading to an increasing demand for specialized caregiving services [1]. As of 2025, in South Korea’s population, the number of people aged 65 years and older exceeds 10.6 million, accounting for over 20% of the total population; the country has already become a super-aged society [2]. Importantly, this pattern is not confined to a single national context. Similar pressures have been observed internationally, alongside projections of rising long-term care demand in OECD countries and Europe [3,4].

However, this rising demand contrasts with the persistent global shortage of care workers. In South Korea, the demand for long-term care will rise from 1.062 million in 2023 to 2.62 million by 2043. To maintain current care ratios, an additional 990,000 caregivers will be required; yet supply is projected to peak at just over 800,000 by the mid-2030s before declining [5]. In response to this workforce deficit, several countries, including Japan and the Nordic countries, have sought to improve labor productivity through the strategic use of digital technologies in care settings [4]. Within this international context, South Korea has increasingly turned to AgeTech (particularly care robots) as a promising technological solution to alleviate human resource constraints and has actively invested in their research and development to address caregiving workforce shortages.

Care robots are gaining attention as a technological solution to support the autonomy of older adults and provide them with more stable and efficient care services [6]. In addition to supporting independence and enhancing the quality of life of older adults, care robots are also expected to reduce the physical and emotional burden of caregivers, thereby contributing to an overall improvement in the quality of care services [7]. At the same time, the use of care robots has raised ethical and social concerns, including potential threats to older adults’ autonomy, risks of dehumanization in care relationships, and privacy issues related to data collection and monitoring [8]. Excessive reliance on robotic care may unintentionally reduce human contact, while continuous sensing technologies can generate concerns about surveillance and data protection. These concerns may shape older adults’ trust and willingness to accept care robots, underscoring the need for human-centered and ethically grounded deployment strategies.

For care robots to be effective, older adults must trust them and actively utilize them in their daily lives. Even though existing studies present positive outcomes regarding the effectiveness of care robots, they often fail to achieve the expected level of effectiveness in real-life situations due to low acceptance among older adults [9,10]. This gap between technological potential and actual use highlights the importance of understanding the factors that shape older adults’ willingness to adopt care robots. In this regard, cross-national studies conducted in Finland, Ireland, and Japan have explored older adults’ perceptions of home-based care robots and identified both shared and context-specific factors explaining their intention to use such technologies [11,12]. These findings underscore the need to move beyond technology-centered approaches and to more closely examine how individual characteristics interact with broader caregiving contexts. However, previous studies have rarely examined the influence of individual characteristic variables on acceptance in a comprehensive manner [13]. Moreover, studies that integrate various factors, including health-related variables, digital competence, and psychological factors, to analyze their combined effects and relative contributions remain comparatively limited [14]. To establish care robots as socially meaningful care services, beyond mere technological innovation, it is essential to identify the key factors influencing older adults’ acceptance and develop strategies that enhance their adoption.

In this context, this study investigates the factors influencing care robot acceptance among older adults in South Korea, employing hierarchical regression analysis (HRA). Variables were entered sequentially: demographic characteristics, health status, digital competence, and technology-related psychological factors. This stepwise approach evaluates each factor group’s relative contributions and explains how attitudes toward digital technology, emotional responses, and confidence in technology use shape acceptance. The findings provide empirical evidence to support care robot adoption and inform policy, service design, and technological innovation for aging populations. Beyond identifying predictors of acceptance, this study contributes to the human–computer interaction literature by integrating functional, digital, and psychological dimensions into a single framework. By highlighting the role of self-efficacy and enthusiasm in shaping older adults’ interaction with care robots, it provides actionable implications for user-centered design and the development of emotionally engaging technologies. These insights can guide policymakers, designers, and service providers in creating care robots that are not only technically functional but also socially and psychologically acceptable. Although this study is situated within the Korean context, it addresses challenges that are increasingly shared across aging societies worldwide. Evidence from a rapidly aging country such as South Korea can offer policy-relevant lessons for other societies facing similar demographic pressures and care workforce constraints [15]. Given the rapid pace of global population aging and the limited empirical evidence on older adults’ acceptance of care robots, the findings of this study provide insights that may inform both research and policy beyond the Korean context.

### 1.1. Theoretical Framework and Analytical Approach

The technology acceptance model (TAM), developed by Davis et al. [16], is a widely recognized theoretical framework for explaining individuals’ acceptance and use of technology. Grounded in the theory of reasoned action (TRA) [17], TAM posits that perceived usefulness and perceived ease of use shape attitudes toward technology, which in turn influence behavioral intention and actual usage.

Subsequent models have extended TAM by incorporating social and emotional dimensions. Among them, the Almere model [18] represents a key theoretical advancement tailored to socially assistive robots for older adults. Building on TAM, the Almere model integrates additional constructs such as trust, perceived sociability, anxiety, enjoyment, and perceived adaptivity, thereby reflecting the interactive, affective, and relational characteristics of care robots. Further refining this approach, Latikka et al. [19] developed a concise care robot acceptance scale that consolidates core constructs from TAM and the Almere model into a single, validated nine-item instrument that is applicable across various care robot types, including assistive, humanoid, telepresence, and companion robots.

Although both TAM and the Almere model conceptualize technology acceptance as a multidimensional construct, the primary objective of this study is to explain overall care robot acceptance rather than to model causal relationships among individual subdimensions. Accordingly, the integrated acceptance scale proposed by Latikka et al. [19] was operationalized as a single composite dependent variable representing general care robot acceptance among older adults. This approach allows for a parsimonious yet theoretically grounded examination of how demographic characteristics, health status, digital competence, and technology-related psychological factors jointly contribute to acceptance.

**Figure 1:**
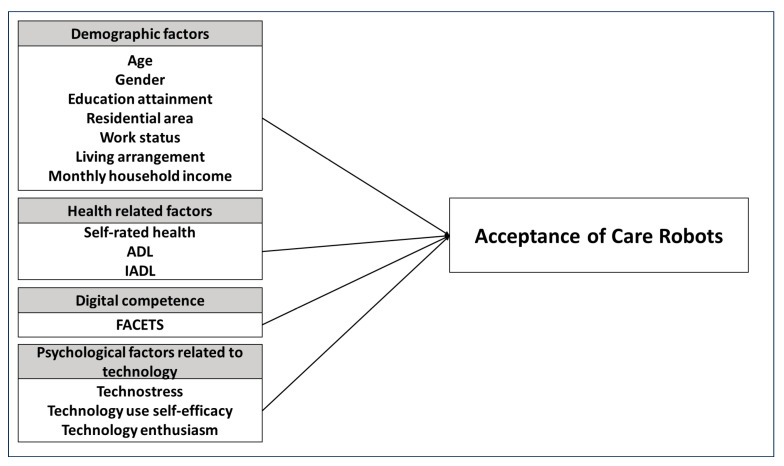
Research model.

Based on this theoretical background, the present study proposes an analytical model in which acceptance of care robots, operationalized using the Latikka et al. [19] scale, is explained by a hierarchically structured set of predictors encompassing demographic characteristics, health status, digital competence, and technology-related psychological factors. The resulting research model is illustrated in Figure 1.

### 1.2. The Relationship Between Demographics and Acceptance of Care Robots

Demographic characteristics, such as age, gender, residential area, employment status, household income, education level, and living arrangement, play a critical role in shaping the acceptance of care robots among older adults. These factors are often among the earliest and most consistent predictors identified in technology adoption studies. Older adults who are younger, economically stable, and highly educated tend to be more receptive to care technologies [20,21,22]. Additionally, Cavallo et al. [23] found that older men were more likely than women to utilize mobility support features when needed and to be receptive to care robot services involving alerts, mobility assistance, and communication functions. Furthermore, Joo et al. [24] indicated that older adults who are employed or live with a spouse or their children are more likely to encounter new technologies; this observation implies that employment status and family cohabitation may positively influence technology acceptance and the intention to use it. Other studies have similarly emphasized the importance of employment status, living arrangements, and residential areas in shaping older adults’ willingness to adopt care technologies [25,26]. Based on these findings, the present study examines demographic characteristics as key contextual factors influencing the acceptance of care robots among older adults.

### 1.3. The Relationship Between Health and Acceptance of Care Robots

The development and deployment of care robots have gained momentum as the number of older adults with physical limitations and care needs continues to rise. Health-related factors play a pivotal role in shaping the acceptance of such technologies, particularly among older adults with greater care requirements. Chen and Chan [20] emphasized that physical, social, and functional health status is a critical determinant of technology acceptance among older adults. Their findings indicated that subjective health positively influenced perceived ease of use but negatively influenced intention to use, whereas cognitive ability, social relationships, attitude toward aging and life satisfaction, and physical functioning had positive effects on intention to use. Building on this work, Chen and Lou [27] combined five elements—self-reported health conditions, cognitive abilities, social relationships, attitude toward aging, and life satisfaction—into a single composite health variable; they found that health positively affected actual technology use.

However, not all health conditions exert the same influence on acceptance. Shin et al. [28] demonstrated that intentions to use daily living assistive technologies varied by health status as determined through frailty. Specifically, older adults in the prefrail group reported higher intentions to use such technologies when their subjective health was poorer, suggesting increased motivation to address unmet care needs. In Taiwan, Chiu et al. [29] observed that individuals with more chronic conditions exhibited greater acceptance of companion robots, indicating that higher health needs may foster openness to robotic care solutions. Therefore, because health-related factors exhibit complex patterns, it is necessary to further examine their influence on the acceptance of care robots. Taken together, prior studies suggest that health-related factors influence care robot acceptance in complex and sometimes contradictory ways. Building on this literature, the present study examines multiple dimensions of health status simultaneously to better understand their roles in shaping acceptance among older adults.

### 1.4. The Relationship Between Digital Competence and Acceptance of Care Robots

Digital competence can be defined as the ability to effectively use technology to enhance and optimize everyday life [30]. It has been widely addressed by scholars and featured in policy discussions. However, over the past few decades, it has at times been examined in conjunction with digital literacy. Studies have shown that digital device use plays an important role in reducing social isolation, improving self-esteem, and promoting participation in social activities [31,32]. Older adults with higher digital literacy tend to have greater access to technology, stronger learning abilities, and higher adaptability, enabling them to respond proactively to changes in the digital environment [33].

Digital competence has emerged as a key determinant of care robot acceptance, as digital technologies are increasingly integrated into care systems. Stafford et al. [34] found that older adults who use robots generally have greater prior experience with computers. Luo et al. [35], in a meta-analysis of factors influencing older adults’ acceptance of social companion robots, identified prior technology experience as a significant predictor. Lee et al. [36] examined the effect of digital literacy on the acceptance of care robot technologies among older adults and confirmed that technology self-efficacy acts as an important mediating factor in this relationship. Their findings indicated that higher digital literacy enhances self-efficacy in care robot use, which in turn increases peoples’ intention to adopt these technologies. Overall, the existing research underscores the importance of digital competence in shaping older adults’ acceptance of care robots. The present study therefore examines digital competence as a predictor of care robot acceptance within an integrative framework.

### 1.5. The Relationship Between Psychological Factors Related to Technology and Acceptance of Care Robots

Technostress refers to the stress individuals experience when struggling to adapt to using information and communication technologies (ICTs) [37]. Cao et al. [38] noted that information overload and excessive system features can induce fatigue and technostress among older users, leading to resistance to technology adoption. In the context of aging populations, technostress undermines users’ confidence and heightens their reluctance to engage with new technologies. For instance, Cha [39] found that technostress significantly reduces older adults’ intention to continue using mobile healthcare applications.

In contrast, self-efficacy, in the technological context, refers to the belief in one’s ability to successfully perform complex technological tasks [40]. Previous studies have consistently shown the positive role of self-efficacy in the adoption of technology. For instance, Latikka et al. [41] found that higher technology use self-efficacy was associated with more positive attitudes toward robots. Similarly, Oh and Choi [42] reported that, among adults aged 65 years and older, higher technology self-efficacy predicted a stronger intention to adopt new technologies; meanwhile, Shin et al. [43] demonstrated that greater self-efficacy in using digital healthcare devices increased older adults’ willingness to use such devices. Together, these findings suggest that, unlike technostress, self-efficacy functions as a key psychological facilitator that encourages technology adoption among older adults.

Furthermore, positive attitudes toward technology among older adults play a significant role in enhancing technology acceptance. Such positive attitudes are often captured by the concept of technology enthusiasm [44], which serves as a key motivator for older adults to more actively embrace and utilize new technologies. Seifert and Schelling [45] demonstrated that affinity for technology positively impacts internet use, with enjoyment and interest during technology use being crucial factors that increase acceptance intention and actual usage behavior. Correspondingly, such enthusiasm has been associated with greater willingness to adopt care robots [21,24]. Collectively, the existing research indicates that psychological factors related to technology, such as technostress, technology self-efficacy, and technology enthusiasm, play a central role in shaping care robot acceptance among older adults. Accordingly, this study examines these factors simultaneously as the most proximal predictors in the hierarchical regression model to assess their relative contributions to care robot acceptance.

This review of the literature underscores the need to comprehensively examine the multiple factors that influence care robot acceptance, providing a clear rationale for the integrative and hierarchical analytical approach adopted in this study.

## 2. Materials and Methods

### 2.1. Data Source and Participants

This study used data from an ongoing research initiative on technology acceptance among older adults in South Korea, conducted by the Department of Gerontology at Kyung Hee University. Initiated in 2022 and conducted biennially, the survey aims to assess the current status of technology use and to examine the needs and acceptance of AgeTech and care robots among older adults.

Specifically, this study utilized data from the first wave of the panel survey conducted in 2022. Between October and November 2022, a face-to-face survey was administered to community-dwelling older adults aged 60 years and older across all provinces in South Korea, which may have facilitated the participation of older adults with limited digital access. The study included a relatively younger-old population to better capture early-stage changes in technology acceptance that tend to emerge during the initial phase of later life, prior to the conventional age threshold of 65 years.

To enhance the demographic representativeness of the sample, a quota sampling method was employed. Quotas were established based on national census data from Statistics Korea, reflecting the distribution of older adults by administrative district. The proportion of participants assigned to each regional category was determined to closely mirror the population distribution of older adults in South Korea. Professionally trained interviewers conducted one-on-one interviews in the assigned survey areas. Surveyors began at designated starting points (e.g., community centers or town offices) indicated on the survey area maps and visited households sequentially. If participants were not available, interviewers made up to three follow-up attempts to contact them.

A total of 507 individuals participated in the survey. After excluding cases with missing data, 506 participants were included in the final analysis. The questionnaire and research procedures were reviewed and approved by the Kyung Hee University Institutional Review Board (KHGIRB-22-468), ensuring ethical compliance and methodological validity. The descriptive characteristics of the study sample (N = 506) are summarized in Table 1.

### 2.2. Measurement

The variables, items, their Cronbach’s alpha values, and descriptive statistics are presented in Table 2.

The dependent variable in this study—the acceptance of care robots—was measured using a nine-item scale developed by Latikka et al. [19], which assesses multiple dimensions of technology acceptance: attitude toward technology, perceived usefulness, perceived ease of use, perceived enjoyment, trust, perceived adaptivity, facilitating conditions, anxiety, and perceived sociability/operating friendliness. The instrument was developed based on Heerink’s items [18] addressing the functional and social acceptance care robots. Each item was rated on a 5-point Likert scale ranging from 1 (totally disagree) to 5 (totally agree). A composite score was computed by summing the responses to all nine items, with higher scores indicating greater acceptance of care robots. The internal consistency of the scale was excellent (Cronbach’s α = 0.93). To enhance respondents’ understanding during the survey, illustrative images and short descriptions of various types of care robots were provided, including those for transferring/lifting, changing body position in bed, feeding, toileting, mobility assistance, bathing, exercising, communication, and smart monitoring or coaching. To minimize potential bias, the images were selected to represent a broad range of care robot types and were presented in a neutral manner, without evaluative descriptions, functional comparisons, or emotional framing.

The independent variables in this study comprised three categories: health-related factors, digital competence, and technology-related psychological factors. Health-related factors included three variables: self-rated health, activities of daily living (ADLs), and instrumental activities of daily living (IADLs). Self-rated health was measured using a single item, “How are your general health conditions?”, rated on a 5-point Likert scale. Higher scores indicate better perceived health. ADLs and IADLs were assessed using a 3-point scale reflecting the level of assistance required for each activity: 1 = completely independent, 2 = some help needed, and 3 = completely dependent [46]. The internal consistency of the ADLs and IADLs scales was high, with Cronbach’s alpha coefficients of 0.82 and 0.89, respectively.

Digital competence was measured using the functional assessment of currently employed technology scale (FACETS) developed by Lepkowsky and Arndt [47]. A variety of instruments have been developed to assess an individual’s perceived proficiency with different technologies. Among these, FACETS is a concise tool that functionally evaluates the frequency of current information technology use across specific functional areas. Moreover, the FACETS highlights individual strengths and gaps in digital engagement by identifying areas where IT use is more or less frequent. The FACETS questionnaire consists of 10 items, with 2 items to represent each of the 5 functional domains: home, social, E-commerce, healthcare, and technical. Each item is rated on a 6-point Likert scale ranging from 0 to 5, reflecting how frequently the respondent employs a specific type of information technology. Higher scores indicate more frequent and extensive utilization of technologies across various domains. The scale demonstrated high internal consistency (Cronbach’s alpha = 0.85).

Psychological factors included technostress, technology use self-efficacy, and technology enthusiasm. Technostress is stress induced by the use of ICT. This study employed the scale developed by Nimrod [48], which has been validated as a reliable instrument for measuring technostress among older adults. Responses were rated on a 4-point Likert scale (1 = not at all; 2 = somewhat disagree; 3 = somewhat agree; 4 = strongly agree), and the total score was calculated by summing the item responses. Higher scores indicated greater technostress. The scale demonstrated acceptable internal consistency, with a Cronbach’s alpha of 0.85. Technology use self-efficacy was measured using four items adapted from Compeau and Higgins [49], Seo and Sung [50], and Lee and Kwon [51]. The items assessed confidence, ease, proficiency, and understanding in using new technologies. Each item was rated on a 5-point Likert scale, and the final score was computed as the average of the four items, with higher scores indicated greater self-efficacy in technology use. The scale demonstrated excellent internal consistency, with a Cronbach’s alpha of 0.93.

Technology enthusiasm, referring to the degree of enjoyment and positive attitude toward using technology, was measured using a scale developed by Anderberg et al. [44], specifically designed to assess attitudes toward technology among older adults. In the present study, perceived enjoyment was operationalized as the enjoyment associated with care robots specifically, whereas technology enthusiasm captures a broader, technology-general disposition. The scale consists of three items, each rated on a 5-point Likert scale. The final score was calculated as the average of the three items, with higher scores indicating greater enthusiasm for technology. The internal consistency of the scale was acceptable, with a Cronbach’s alpha of 0.73.

To assess potential common method bias, Harman’s single-factor test was conducted using principal component analysis with orthogonal Varimax (Horst) rotation. The results showed that a single factor accounted for 19.43% of the total variance, which is well below the 50% threshold; this suggests that common method bias is unlikely to be a serious concern. Confirmatory factor analysis (CFA) was conducted as a supplementary analysis (Table S1) for key multi-item constructs with sufficient degrees of freedom, including digital competence (FACETS), technostress, and acceptance of care robots. Technology-related psychological constructs with limited degrees of freedom were assessed using internal consistency reliability indices (Cronbach’s alpha) rather than CFA.

### 2.3. Analytical Strategy

First, descriptive statistics were calculated to examine the characteristics of the participants with respect to key variables, including demographics (e.g., age, gender, educational attainment, residential area, work status, living arrangement, and monthly household income), health-related factors, digital competence, and psychological factors related to technology. Frequencies and percentages were reported for categorical variables, while means and standard deviations were reported for continuous variables. Second, bivariate Pearson correlation analysis was conducted to assess the linear relationships between independent variables and examine multicollinearity. Finally, hierarchical multiple regression analysis was performed to identify the predictors of care robot acceptance. hierarchical regression analysis enables the sequential entry of conceptually distinct variable blocks, allowing for the assessment of the incremental explanatory power of each factor group. Compared to alternative approaches, such as structural equation modeling, HRA was deemed more appropriate given the study’s focus on comparative explanatory contributions rather than testing a fully specified causal model among the latent constructs. In step one, demographic variables were entered. In step two, health-related variables, such as self-rated health and functional ability (ADLs and IADLs), were added. Step three included digital competence. In the final step, psychological factors related to technology, namely technostress, technology use self-efficacy, and technology enthusiasm, were added. This stepwise approach allowed for the incremental examination of the explanatory power of each group of variables. In addition, moderation analyses were conducted to determine whether digital competence moderates the relationships between technology-related psychological factors and care robot acceptance. Interaction terms were created using mean-centered variables to reduce multicollinearity, and significant interaction effects were further examined using simple slope analyses.

All statistical analyses were conducted using Stata version 17.0 (StataCorp LLC, College Station, TX, USA). Statistical significance was set at *p* < 0.05.

## 3. Results

### 3.1. Correlation Analyses

A Pearson correlation analysis was conducted using Stata 17.0. All correlations were found to occur in the expected direction; none exceeded 0.80, suggesting that multicollinearity was not a significant issue among independent variables. The detailed correlation matrix is presented in Table 3.

### 3.2. Hierarchical Regression Analyses

Table 4 presents the results of the hierarchical regression analysis conducted to predict the acceptance of care robots among older adults. The model controlled demographic variables (age, gender, educational attainment, residential area, work status, living arrangement, and monthly household income) and sequentially added health-related factors (self-rated health, ADLs, and IADLs), digital competence, and technology-related psychological factors (technostress, technology use self-efficacy, and technology enthusiasm). In step 1, demographic variables were found to explain 19% of the variance in care robot acceptance. Significant predictors included age (β = −0.12, *p* < 0.05), educational attainment (β = 0.23, *p* < 0.001), residential area (β = 0.12, *p* < 0.01), and monthly household income (β = 0.19, *p* < 0.01). In step 2, the inclusion of health-related factors increased the explained variance to 23%, yielding an additional 4% (ΔR^2^ = 0.04), which corresponds to a small-to-moderate effect size. The effect of self-rated health (β = 0.15, *p* < 0.01) was significant, while the effect of age was no longer significant. In step 3, digital competence (measured through the FACETS score) was added and showed a significant association with care robot acceptance (β = 0.17, *p* < 0.01). However, the explained variance increased only marginally to 24% (ΔR^2^ = 0.01), indicating a small effect size. At this step, work status (β = −0.10, *p* < 0.05) became significant, whereas monthly household income lost its significance. In step 4, the inclusion of technology-related psychological factors substantially improved the model, increasing the total explained variance to 41%. The additional 17% increase in explained variance (ΔR^2^ = 0.17) represents a large effect size, highlighting the strong contribution of psychological factors related to technology. Technology use self-efficacy (β = 0.28, *p* < 0.001) and technology enthusiasm (β = 0.28, *p* < 0.001) significantly predicted care robot acceptance. Among previously significant variables, only IADLs remained significant (β = −0.10, *p* < 0.05), while the effects of other demographic, health-related, and digital competence variables were no longer observed. The magnitude of standardized regression coefficients was interpreted using conventional benchmarks suggested by Cohen [52], with values of approximately 0.10, 0.30, and 0.50 indicating small, medium, and large effects, respectively. Changes in explained variance (ΔR^2^) were interpreted using established guidelines, where values of approximately 0.01, 0.09, and 0.25 were considered small, medium, and large effect sizes, respectively [52,53].

Taken together, these findings suggest that, while demographic and health-related characteristics play a role in care robot acceptance, psychological readiness for technology—particularly confidence in using technology and positive emotional engagement—has the greatest practical importance. The results imply that efforts to promote care robot adoption among older adults may benefit more from interventions that strengthen technology self-efficacy and enthusiasm, rather than focusing solely on demographic targeting or physical health status.

### 3.3. The Moderating Role of Digital Competence

To further examine the joint effects of psychological and skill-based factors, moderation analyses were conducted (Table 5). Digital competence did not significantly moderate the relationship between technology use self-efficacy and care robot acceptance, indicating that the effect of self-efficacy was consistent across different levels of digital competence.

In contrast, a significant interaction was found between technology enthusiasm and digital competence. The interaction term was negative and statistically significant (β = −0.09, *p* < 0.05), indicating that the positive association between technology enthusiasm and care robot acceptance weakened as digital competence increased.

**Figure 2:**
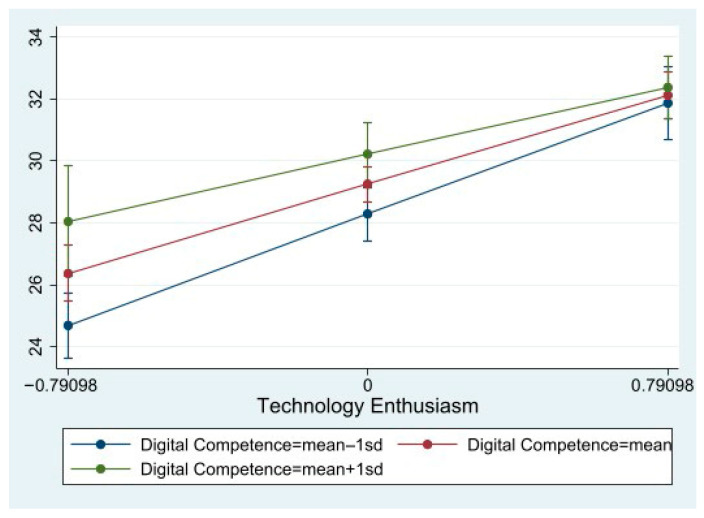
Moderating Role of Digital Competence in the Relationship between Technology Enthusiasm and Care Robot Acceptance.

The simple slope analyses (Figure 2) further showed that technology enthusiasm had a stronger positive effect on care robot acceptance among older adults with lower levels of digital competence (β = 0.41, *p* < 0.001), whereas this effect was attenuated among those with higher digital competence (β = 0.23, *p* < 0.001).

## 4. Discussion

This study examined factors influencing care robot acceptance among older adults in South Korea using a hierarchical regression approach, sequentially incorporating demographic, health-related, digital competence, and technology-related psychological variables. This integrative framework allowed for a comprehensive assessment of the determinants of the adoption of technology among an aging population.

Demographic factors initially predicted greater acceptance of care robots among younger, more educated, and urban-dwelling participants with higher household income; this finding is consistent with those of previous studies, highlighting the structural and socioeconomic barriers to technology use among older adults [20,21,25,26]. However, in the final model, their predictive power disappeared once health-related, digital competence, and technology-related psychological variables were included, indicating that demographic characteristics function more as contextual enablers than decisive determinants. These factors provide foundational access and exposure to technology, facilitating opportunities for engagement, but they do not directly drive acceptance in the presence of stronger functional and psychological predictors.

While some studies have suggested that older adults with poorer health may adopt technology to compensate for functional limitations [28,29], this study found that better self-rated health and higher IADLs were associated with greater care robot acceptance. In other words, older adults who perceive themselves as healthy and independent are more receptive to new technologies and view care robots not as symbols of dependency but as tools for maintaining autonomy. From this perspective, care robots are not merely technologies used out of necessity; instead, they are tools chosen to enhance quality of life—this notion is particularly meaningful for those who are healthy and functionally independent. Supporting this interpretation, Doba et al. [54] reported that lower IADLs ability is associated with reduced self-efficacy in daily life, which in turn reduces confidence in learning and using new technologies [55]. Consequently, older adults with stronger IADLs abilities are more likely to perceive care robots as instruments that improve efficiency and convenience in daily life, and their greater confidence in technology use translates into more positive attitudes toward adoption. These findings suggest that strategies to promote care robot adoption should target not only older adults with high care needs but also those who are relatively healthy and independent. Tailoring care robot design and communication to emphasize their role in supporting autonomy, prolonging independence, and preventing functional decline may be especially effective for Korean older adults.

Digital competence predicted care robot acceptance prior to the inclusion of psychological variables, indicating that technological skills form a foundation for interacting with digital tools. Greater competence enhances access, understanding, and effective use, facilitating initial engagement with care robots [36,56]. However, its influence diminished once factors such as technology self-efficacy and technology enthusiasm were considered. This suggests that even individuals with sufficient digital competence ultimately rely more on their confidence and positive attitudes toward technology when deciding whether to adopt care robots.

Technology-related psychological factors, particularly technology use self-efficacy and technology enthusiasm, emerged as the strongest predictors of care robot acceptance, outweighing demographic, health, and digital competence factors. These findings align with previous studies, showing that higher technology self-efficacy strengthens older adults’ intention to adopt new technologies [42,43] and that technology enthusiasm is associated with greater willingness to adopt care robots [21,24]. In other words, even when older adults possess adequate digital competence or favorable health, their willingness to accept care robots largely depends on their confidence in using technology and their enthusiasm for it. Although technostress was included in the model, it was not a significant predictor, possibly because its negative effects are overshadowed by the stronger positive influences of self-efficacy and enthusiasm, or its impact operates indirectly through these psychological factors rather than as a direct determinant. The moderation analyses further clarified the joint effects of psychological and skill-based factors. Digital competence did not significantly moderate the relationship between technology use self-efficacy and care robot acceptance, indicating that the positive effect of self-efficacy was robust across different levels of digital competence. This suggests that confidence in using technology functions as a universally important psychological resource for older adults, regardless of their actual skill level. In contrast, a significant interaction was observed between technology enthusiasm and digital competence. Technology enthusiasm exerted a stronger influence on acceptance among older adults with lower levels of digital competence, whereas its effect was attenuated among those with higher digital competence. This pattern suggests that enthusiasm plays a compensatory role for individuals with limited digital skills, sustaining acceptance despite lower competence; meanwhile, among digitally competent individuals, acceptance may be less dependent on affective motivation alone.

Although technostress was included in the model, it did not emerge as a significant predictor of care robot acceptance. This non-significant finding may be explained by the predominance of positive psychological factors, such as self-efficacy and enthusiasm, which appear to outweigh stress-related concerns in voluntary technology adoption contexts. Furthermore, technostress may operate indirectly—by undermining self-efficacy or dampening enthusiasm—rather than exerting a direct effect. Previous studies have indicated that positive affect can mediate the relationship between technological stressors and AI adoption intention [57]. This suggests that the impact of technostress may be mediated or moderated by internal psychological resources; this is a possibility that warrants further investigation in future research.

The short-term practical and policy implications of these findings emphasize the design and implementation of programs that strengthen self-efficacy and technology enthusiasm through hands-on, experiential learning. Strategies to strengthen self-efficacy include providing repeated small success experiences and offering real-time feedback and emotional encouragement. Engagement in and maintenance of online activities have also been shown to positively influence attitudes towards and enthusiasm for technology [58]. For instance, interventions may operate along two complementary tracks: a standard track of approximately four weeks with weekly 60–90 min sessions focusing on practical, scenario-based learning (including routine support, safety monitoring, connectivity, and cognitive or leisure activities); an intensive track of around two weeks, incorporating 1:1 or 1:2 micro-coaching for participants with low confidence or initial reluctance, and providing a high density of successful experiences to rapidly enhance self-efficacy and engagement. Follow-up support during the initial adaptation period of two–four weeks is recommended to prevent drop-off, including simple checks, problem-solving assistance, and refresher activities. User-centered robot design should complement these interventions by incorporating adaptive interfaces, real-time feedback, positive reinforcement, personalized interaction, and mechanisms to sustain enthusiasm, particularly for users with lower digital competence. Beyond providing practical support, robots should also facilitate meaningful social and emotional engagement, addressing older adults’ broader well-being [59]. Leveraging existing national infrastructures, such as digital learning centers and senior welfare centers, can facilitate program implementation. Seoul’s Digital Action Plaza, for example, provides public access to AI, robots, and kiosks; expanding such facilities to include care robot modules would provide repeated practical experiences rather than lecture-based instruction, supporting accessibility and scalability in real-world settings.

While this study provides important insights into the psychological factors influencing care robot adoption, long-term research and societal implications point to opportunities for further exploration and validation. The cross-sectional design precludes causal inference, and the study measured acceptance intentions rather than actual robot use, limiting understanding of how older adults’ attitudes translate into sustained engagement. Additionally, prior experience with robots and digital technologies was not fully accounted for, affecting generalizability. Future research should employ longitudinal or experimental designs to examine how self-efficacy, enthusiasm, and acceptance evolve with actual robot exposure and usage, including whether repeated interactions and maintenance support reinforce or modify initial attitudes. Investigating mediating and moderating relationships among psychological, health, and digital competence variables using structural equation modeling or multilevel analyses will provide insight into the mechanisms that shape adoption. Expanding samples to diverse regions, care needs, and levels of technology access will further enhance generalizability and inform sustainable integration of care robots into society.

Overall, despite these limitations, this study demonstrates that technology-related psychological factors, particularly self-efficacy and enthusiasm, play pivotal roles in promoting care robot adoption among older adults. The findings offer actionable guidance for designing interventions, user-centered robot development, and policy strategies aimed at enhancing digital inclusion, autonomy, and sustained engagement with AgeTech solutions, balancing immediate practical impact with long-term research and societal considerations.

## 5. Conclusions

In the context of a super-aged society, care robots and AgeTech offer strategic solutions to support older adults’ autonomy and quality of life. This study highlights the pivotal role of technology-related psychological factors, particularly technology use self-efficacy and technology enthusiasm, in relation to care robot acceptance among older adults. The results suggest that older adults’ confidence and positive attitudes toward technology may play key roles in shaping acceptance, beyond demographic or health characteristics. The study has several limitations, including its cross-sectional design; reliance on acceptance intentions rather than actual robot use; and potential sampling biases in regional, urban–rural, and age-group representation. Future research should employ longitudinal or experimental designs to examine how self-efficacy and enthusiasm evolve with real-world robot exposure, and explore mediating and moderating mechanisms among psychological, health, and digital competence factors. Despite these limitations, the findings provide actionable insights for designing interventions, user-centered robot development, and policy strategies aimed at enhancing digital inclusion, autonomy, and sustained engagement with AgeTech solutions among older adults.

## Figures and Tables

**Table 1:** Sample’s descriptive characteristics (n = 506).

Characteristics	Mean (Range)/n	SD/%
Age	70.11 (60–93)	7.74
Gender		
Men	276	54.55
Women	230	45.45
Educational attainment		
No formal education	34	6.72
Elementary school	127	25.10
Middle school	106	20.95
High school	191	37.75
College or above	48	9.49
Residential area		
Urban	388	76.68
Rural	118	23.32
Work status		
Employed	265	52.37
Not employed	241	47.63
Living arrangement		
Living alone	132	26.09
Not living alone	374	73.91
Monthly household income ^a^	266.85 (30–1000)	196.50

Note: ^a^ The monthly household income was measured at KRW 10,000, which was equivalent to USD 7.20 in 2025.

**Table 2:** Measurement variables, items, Cronbach’s alpha, and descriptive statistics.

Variables	Cronbach’s Alpha	M (Range)	SD
Dependent variable			
Acceptance of care robots			
I think it’s a good idea to use the care robot (attitude towards technology)	0.93	28.94 (9–45)	7.01
I think the care robot would be useful in my job (perceived usefulness)			
I think I can use the care robot without any help (perceived ease of use)			
Working with the care robot would be pleasant (perceived enjoyment)			
I would not be worried about the safety of using the care robot (trust)			
I think the care robot can be adapted to what I need (perceived adaptivity)			
I know enough of the care robot to make good use of it (facilitating conditions)			
I would not be afraid to make mistakes with the care robot (anxiety)			
Pleasant/smooth interaction (perceived sociability/operating friendliness)			
Independent variables			
1. Health-related factors			
1.1. Self-rated health			
How are your general health conditions?	-	3.39 (1–5)	0.93
1.2. Activities of Daily Living (ADLs)			
Dressing	0.82	13.10 (13–22)	0.67
Face washing			
Brushing teeth			
Shampooing hair			
Grooming: nail care			
Eating			
Changing position			
Sitting up from lying position			
Transferring			
Using the toilet			
Bowel control			
Bladder control			
Bathing or showering			
1.3. Instrumental Activities of Daily Living (IADLs)			
Doing housework or handyman work	0.89	10.66 (10–27)	1.98
Food preparation			
Laundry			
Managing money			
Grocery shopping			
Ability to use telephone			
Using transportation			
Getting to places beyond walking distance			
Grooming			
Taking medications			
2. Digital Competence			
2.1 Functional Assessment of Currently Employed Technology Scale (FACETS)			
Send email	0.85	8.58 (0–50)	8.74
Find, open & close files in my computer			
Find, open & close files in my computer			
Post on social media (e.g., Facebook, Twitter)			
Manage my banking and credit card accounts online			
Pay bills and make purchases via the internet			
Communicate with my doctor or clinic online			
Communicate with my health insurance company online			
Install components (monitors, speakers, mice)			
Reset a modem or router in my home			
3. Psychological factors related to technology			
3.1 Technostress			
This technology makes me do things slower	0.80	2.61 (1.50–3.79)	0.36
This technology makes me respond more quickly than I would normally do ^a^			
This technology creates many more problems than I would otherwise experience			
Using this technology blurs boundaries between my out-of-home and my home life			
I feel my personal life is being interrupted by this technology			
I often find the technology too complex to use			
I do not know enough about this technology to use it effectively			
The constant developments and upgrades in the technology are a burden for me			
I feel uncomfortable that my use of this technology can be easily monitored			
It bothers me that the information created by my current technology use could be traced even years from now			
I feel that my use of this technology makes it easier to invade my privacy			
I am better at understanding and using technology than young people ^a^			
I am typically behind younger persons in my family in the technology I use			
If young people are residents in ‘technology-land,’ I may be considered an immigrant			
3.2 Technology use Self-efficacy			
I am confident in using new technologies.	0.93	3.19 (1–5)	1.01
I do not have difficulties in using new technologies.			
I can use new technologies proficiently.			
I understand how to use new technologies.			
3.3 Technology enthusiasm			
I think it’s fun with new technological gadgets	0.73	3.09 (1–5)	0.79
Using technology makes life easier for me			
I like to acquire the latest models or updates			

Note. ^a^ Reverse-scored results.

**Table 3:** Results of correlation analysis.

	1	2	3	4	5	6	7	8
1. Acceptance of care robot	-							
2. Self-rated health	0.28 ***	-						
3. ADLs	−0.11 *	−0.17 ***	-					
4. IADLs	−0.21 ***	−0.26 ***	0.62 ***	-				
5. Digital competence	0.39 ***	0.32 ***	−0.10 *	−0.16 ***	-			
6. Technostress	−0.24 ***	−0.12	−0.11 *	−0.09	−0.29 ***	-		
7. Technology use self-efficacy	0.56 ***	0.32 ***	−0.08	−0.12 **	0.53 ***	−0.33 ***	-	
8. Technology enthusiasm	0.56 ***	0.30 ***	−0.07	−0.18 ***	0.44 ***	−0.26 ***	0.66 ***	-

Note. * *p* < 0.05; ** *p* < 0.01; *** *p* < 0.001.

**Table 4:** Results of hierarchical regression analysis.

	Model 1				Model 2				Model 3				Model 4			
	β	95% CI		t	β	95% CI		t	Β	95% CI		t	β	95% CI		t
		Low	High			Low	High			Low	High			Low	High	
Step 1. Demographics																
Age	−0.12	−0.20	−0.02	−2.42 *	−0.08	−0.16	0.01	−1.70	0.04	−0.13	0.05	−0.90	−0.05	−0.06	0.10	0.41
Gender	0.02	−0.86	1.57	0.57	0.04	−0.65	1.74	0.89	−0.05	−0.66	1.71	0.86	0.02	−1.76	0.37	−1.28
Educational attainment	0.23	0.70	1.78	4.48 ***	0.20	0.54	1.61	3.93 ***	0.13	0.13	1.29	2.41 *	0.09	−0.04	0.99	1.80
Residential area	0.12	0.63	3.39	2.86 **	0.10	0.34	3.07	2.45 *	0.09	0.18	2.90	2.23 *	0.03	−0.66	1.76	0.89
Work status	−0.04	−1.88	0.68	−0.92	−0.09	−2.52	0.06	−1.88	−0.10	−2.62	−0.07	−2.07 *	−0.05	−1.85	0.42	−1.24
Living arrangement	0.06	−0.82	2.60	1.02	0.04	−1.12	2.26	0.66	0.01	−1.58	1.82	0.14	0	−1.55	1.45	−0.07
Monthly household income	0.19	0.57	2.70	3.02 **	0.16	0.45	2.56	2.80 **	0.12	−0.03	2.13	1.91	0.08	−0.25	1.67	1.46
Step 2. Health-related factors																
Self-rated health					0.15	0.41	1.71	3.21 **	0.13	0.33	1.62	2.96 **	0.04	−0.26	0.90	1.08
ADLs					−0.02	−1.24	0.86	−0.36	−0.01	−1.12	0.97	−0.14	−0.01	−1.07	0.78	−0.30
IADLs					−0.09	−0.73	0.01	−1.94	−0.11	−0.75	−0.02	−2.08 *	−0.10	−0.67	−0.02	−2.08 *
Step 3. Digital Competence																
FACETS									0.17	0.05	0.23	3.14 **	0.01	−0.08	0.09	0.12
Step 4. Psychological factors related to technology																
Technostress													−0.05	−2.41	0.45	−1.34
Technology use Self-efficacy													0.28	1.24	2.59	5.60 ***
Technology Enthusiasm													0.28	1.64	3.31	5.85 ***
R^2^ (Adj. R^2^)	0.19 (0.18)				0.23 (0.21)				0.24 (0.23)				0.41 (0.40)			
R^2^ change					0.03 ***				0.02 ***				0.17 ***			
F	17.18 ***				14.66 ***				14.46 ***				24.91 ***			
F change					7.45 ***				9.28 **				47.60 ***			

Note. N = 506. * *p* < 0.05; ** *p* < 0.01; *** *p* < 0.001. VIF results are provided in Table S2.

**Table 5:** Moderating Effect of Digital Competence on the Relationship between Technology Enthusiasm and Care Robot Acceptance.

	β	95% CI		t
		Low	High	
Technology Enthusiasm	0.41	2.86	4.42	9.20 ***
Digital Competence	0.14	0.02	0.20	2.47 *
Technology Enthusiasm * Digital Competence	−0.09	−0.19	−0.01	−2.25 *
Age	−0.01	−0.09	0.07	−0.22
Gender	−0.03	−1.51	0.69	−0.73
Educational attainment	0.10	0.02 *	1.09	2.04
Residential area	0.06	−0.32	2.17	1.47
Work status	−0.04	−1.65	0.64	−0.87
Living arrangement	0.02	−1.29	1.80	0.32
Monthly household income	0.09	−0.21	1.76	1.55
Constant		15.08	32.24	5.42 ***
R^2^ (Adj. R^2^)	0.37 (0.35)			
F	28.64 ***			

Note. N = 506. * *p* < 0.05; *** *p* < 0.001.

## Data Availability

The data presented in this study are available on request from the corresponding author due to ethical restrictions and privacy concerns related to the protection of sensitive personal information collected from older adult participants.

## Supplementary Materials

The following supporting information can be downloaded at: https://www.mdpi.com/article/10.3390/healthcare14030322/s1, Table S1. Confirmatory Factor Analysis. Table S2. Variance Inflation Factors (VIFs) result.

## References

[1] OECD (2023). Health at a Glance 2023: OECD Indicators.

[2] Statistics Korea (2025). Search Results for “Elderly Population”. KOSIS Korean Statistical Information Service. https://kosis.kr/search/search.do?query=%EB%85%B8%EC%9D%B8%EC%9D%B8%EA%B5%AC.

[3] European Commission, Directorate-General for Employment, Social Affairs and Inclusion Long-Term Care. https://employment-social-affairs.ec.europa.eu/policies-and-activities/social-protection-social-inclusion/social-protection/long-term-care_en.

[4] OECD (2024). Is Care Affordable for Older People?.

[5] The Presidential Committee on Ageing Society and Population Policy (2025). The 11th Emergency Population Strategy Meeting.

[6] Padhan S., Mohapatra A., Ramasamy S.K., Agrawal S. (2023). rtificial intelligence (AI) and robotics in elderly healthcare: Enabling independence and quality of life. Cureus.

[7] Kim Y.S., Shin H.R., Yoon H.J., Ban S.W., Jung K.W., In H. (2024). Usability study of a smart transfer-assistive robot with dual arms for care workers. Disabil. Rehabil. Assist. Technol..

[8] Park K., Park Y.O. (2025). A Sociotechnical Perspective on the Use of Care Robots in Healthcare Services: A Scoping Review. J. Korean Soc. Living Environ. Syst..

[9] Beer J.M., Prakash A., Smarr C.-A., Chen T.L., Hawkins K., Nguyen H., Deyle T., Mitzner T.L., Kemp C.C., Rogers W.A. (2017). Older Users’ Acceptance of an Assistive Robot: Attitudinal Changes Following Brief Exposure. Gerontechnology.

[10] Macalupu V., Miller E., Martin L., Caldwell G. (2025). Human–robot interactions and experiences of staff and service robots in aged care. Sci. Rep..

[11] Ide H., Suwa S., Akuta Y., Kodate N., Tsujimura M., Ishimaru M., Shimamura A., Kitinoja H., Donnelly S., Hallila J. (2024). A comparative study to elucidate factors explaining willingness to use home-care robots in Japan, Ireland, and Finland. Sci. Rep..

[12] Suwa S., Tsujimura M., Kodate N., Donnelly S., Kitinoja H., Hallila J., Toivonen M., Ide H., Bergman-Kärpijoki C., Takahashi E. (2020). Exploring perceptions toward home-care robots for older people in Finland, Ireland, and Japan: A comparative questionnaire study. Arch. Gerontol. Geriatr..

[13] Liu J., Wang X., Zhang J. (2025). Investigating Elderly Individuals’ Acceptance of Artificial Intelligence (AI)-Powered Companion Robots: The Influence of Individual Characteristics. Behav. Sci..

[14] Abdi J., Al-Hindawi A., Ng T., Vizcaychipi M.P. (2018). Scoping review on the use of socially assistive robot technology in elderly care. BMJ Open.

[15] Kim H., Kwon S. (2021). A decade of public long-term care insurance in South Korea: Policy lessons for aging countries. Health Policy.

[16] Davis F.D., Bagozzi R.P., Warshaw P.R. (1989). Technology acceptance model. J. Manag. Sci..

[17] Fishbein M., Ajzen I. (1977). Belief, attitude, intention, and behavior: An introduction to theory and research. Philos. Rhetor..

[18] Heerink M., Kröse B., Evers V., Wielinga B. (2010). Assessing acceptance of assistive social agent technology by older adults: The almere model. Int. J. Soc. Robot..

[19] Latikka R., Turja T., Oksanen A. (2019). Self-efficacy and acceptance of robots. Comput. Hum. Behav..

[20] Chen K., Chan A.H.S. (2014). Gerontechnology acceptance by elderly Hong Kong Chinese: A senior technology acceptance model (STAM). Ergonomics.

[21] He Q., He Y., Liu Q., Ma C. (2023). Acceptance of social assistant robots for the older adults living in the community in China. Geriatr. Nurs..

[22] De Veer A.J.E., Peeters J.M., Brabers A.E.M., Schellevis F.G., Rademakers J.J.D.J.M., Francke A.L. (2015). Determinants of the intention to use e-Health by community dwelling older people. BMC Health Serv. Res..

[23] Cavallo F., Esposito R., Limosani R., Manzi A., Bevilacqua R., Felici E., Di Nuovo A., Cangelosi A., Lattanzio F., Dario P. (2018). Robotic services acceptance in smart environments with older adults: User satisfaction and acceptability study. J. Med. Internet Res..

[24] Susanna J., Bomi C., Heyjung U. (2021). Associated factors of technology acceptance attitude and usage intention for wearable robots among older adults. Korean J. Secur. Converg. Manag..

[25] Berkowsky R.W., Sharit J., Czaja S.J. (2017). Factors predicting decisions about technology adoption among older adults. Innov. Aging.

[26] Etemad-Sajadi R., Dos Santos G.G. (2019). Senior citizens’ acceptance of connected health technologies in their homes. Int. J. Health Care Qual. Assur..

[27] Chen K., Lou V.W.Q. (2020). Measuring senior technology acceptance: Development of a brief, 14-item scale. Innov. Aging.

[28] Shin H.R., Um S.R., Yoon H.J., Choi E.Y., Shin W.C., Lee H.Y., Kim Y.S. (2023). Comprehensive senior technology acceptance model of daily living assistive technology for older adults with frailty: Cross-sectional study. J. Med. Internet Res..

[29] Chiu C.-J., Hsieh S., Li C.-W. (2021). Needs and preferences of middle-aged and older adults in Taiwan for companion robots and pets: Survey study. J. Med. Internet Res..

[30] Instituto Nacional de Tecnologías Educativas y de Formación del Profesorado (INTEF) (2017). Marco Común de Competencia Digital Docente.

[31] Yang P., Bi G., Qi J., Wang X., Yang Y., Xu L. (2025). Multimodal wearable intelligence for dementia care in healthcare 4.0: A survey. Inf. Syst. Front..

[32] Xie L., Chen S., Shang Y., Song D. (2024). The effect of internet use behaviors on loneliness: The mediating role of real-life interpersonal communication. Curr. Psychol..

[33] Arcury T.A., Sandberg J.C., Melius K.P., Quandt S.A., Leng X., Latulipe C., Miller D.P., Smith D.A., Bertoni A.G. (2020). Older Adult Internet Use and eHealth Literacy. J. Appl. Gerontol..

[34] Stafford R.Q., MacDonald B.A., Jayawardena C., Wegner D.M., Broadbent E. (2014). Does the robot have a mind? Mind perception and attitudes towards robots predict use of an eldercare robot. Int. J. Soc. Robot..

[35] Luo C., Yuan R., Mao B., Liu Q., Wang W., He Y. (2024). Technology Acceptance of Socially Assistive Robots Among Older Adults and the Factors Influencing It: A Meta-Analysis. J. Appl. Gerontol..

[36] Lee J.W., Cha E.G., Lee H.J., Shin H.R., Kim Y.S. (2024). Impact of Digital Literacy of older adults on Acceptance of Care Robot Technology: Focusing on the Mediating Effect of Technology Self-Efficacy. J. Inf. Syst..

[37] Tarafdar M., Tu Q., Ragu-Nathan B.S., Ragu-Nathan T.S. (2007). The impact of technostress on role stress and productivity. J. Manag. Inf. Syst..

[38] Cao Y., Li J., Qin X., Hu B. (2020). Examining the effect of overload on the mHealth application resistance behavior of elderly users: An SOR perspective. Int. J. Environ. Res. Public Health.

[39] Cha J. (2021). The Effect of UI Usability of Mobile Healthcare Applications on Technostress and Continuous Use Intention: Focusing on Elderly Users. J. Digit. Converg..

[40] McDonald T., Siegall M. (1992). The effects of technological self-efficacy and job focus on job performance, attitudes, and withdrawal behaviors. J. Psychol..

[41] Latikka R., Savela N., Koivula A., Oksanen A. (2021). Attitudes toward robots as equipment and coworkers and the impact of robot autonomy level. Int. J. Soc. Robot..

[42] Oh S.M., Choi S.S. (2021). The Effect of Digital Information Level on the Intention to Use New Technology among Older Adults: Focused on the Multiple Mediating Effect of Technical Self-Efficacy and Utilization Performance. Korean J. Gerontol. Soc. Welf..

[43] Shin H.R., Kim S.K., Kim Y.S. (2020). Effect of self-efficacy of middle-aged and elderly on the intention to use digital health devices focusing on the difference between middle-aged and elderly. J. Digit. Converg..

[44] Anderberg P., Eivazzadeh S., Berglund J.S. (2019). A novel instrument for measuring older people’s attitudes toward technology (TechPH): Development and validation. J. Med. Internet Res..

[45] Seifert A., Schelling H.R. (2018). Seniors online: Attitudes toward the internet and coping with everyday life. J. Appl. Gerontol..

[46] Ministry of Health and Welfare, National Health Insurance Service (2022). Long-Term Care Accreditation Ques-Tionnaire (Appendix Form No. 5) [Questionnaire]. https://www.longtermcare.or.kr/npbs/d/m/000/moveBoardView?menuId=npe0000000920&bKey=B0017&search_boardId=60073.

[47] Lepkowsky C.M., Arndt S. (2018). Functional Assessment of Currently Employed Technology Scale (FACETS): Reliability and Validity. Int. J. Med. Sci. Clin. Inven..

[48] Nimrod G. (2018). Technostress: Measuring a new threat to well-being in later life. Aging Ment. Health.

[49] Compeau D.R., Higgins C.A. (1995). Computer self-efficacy: Development of a measure and initial test. MIS Q..

[50] Suh C.K., Seong S.J. (2004). Individual Characteristics Affecting User’s Intention to Use Internet Shopping Mall. J. MIS Res..

[51] Lee H.B., Kwon N.K. (2006). The Role of Internet Self-efficacy in Internet Shopping. Asia Mark. J..

[52] Cohen J. (1988). Statistical Power Analysis for the Behavioral Sciences.

[53] Aguinis H., Beaty J.C., Boik R.J., Pierce C.A. (2005). Effect size and power in assessing moderating effects of categorical variables using multiple regression: A 30-year review. J. Appl. Psychol..

[54] Doba N., Tokuda Y., Saiki K., Kushiro T., Hirano M., Matsubara Y., Hinohara S. (2016). Assessment of self-efficacy and its relationship with frailty in the elderly. Intern. Med..

[55] Rahman M.S., Ko M., Warren J., Carpenter D. (2016). Healthcare Technology Self-Efficacy (HTSE) and its influence on individual attitude: An empirical study. Comput. Hum. Behav..

[56] Lee H., Chung M.A., Kim H., Nam E.W. (2022). The effect of cognitive function health care using artificial intelligence robots for older adults: Systematic review and meta-analysis. JMIR Aging.

[57] Chang P.-C., Zhang W., Cai Q., Guo H. (2024). Does AI-driven technostress promote or hinder employees’ artificial intelligence adoption intention? A moderated mediation model of affective reactions and technical self-efficacy. Psychol. Res. Behav. Manag..

[58] Berner J., Dallora A.L., Palm B., Berglund J.S., Anderberg P. (2023). Five-factor model, technology enthusiasm and technology anxiety. Digit. Health.

[59] Zguda P., Radosz-Knawa Z., Kukier T., Radosz M., Kamińska A., Indurkhya B. (2025). How Do Older Adults Perceive Technology and Robots? A Participatory Study in a Care Center in Poland. Electronics.

