# Supplementary material for: An Integrated Analysis of Factors Influencing Acceptance of Care Robots Among Older Korean Adults
Source: Healthcare (Basel). 2026 Jan 27;14(3):322. doi: 10.3390/healthcare14030322 (PMC12897706; doi:10.3390/healthcare14030322)
Supplement: Supplementary file 1 [file healthcare-14-00322-s001.zip › healthcare-4090624-supplementary.pdf]

Table S1. Confirmatory Factor Analysis

	χ^2(p)	df	RMSEA	SRMR	CFI	TLI
**Functional Assessment of Currently Employed Technology Scale**	98.110(<0.001)	25	0.076	0.033	0.977	0.958
**Technostress**	317.138(<0.001)	67	0.086	0.088	0.877	0.833
**Acceptance of care robots**	239.804(<0.001)	27	0.125	0.050	0.931	0.908

Table S2. Variance Inflation Factors (VIFs) result

	**Model 1**	**Model 2**	**Model 3**	**Model 4**
**Age**	1.51	1.57	1.66	1.70
**Gender**	1.19	1.20	1.20	1.25
**Educational attainment**	1.57	1.60	1.89	1.90
**Residential area**	1.12	1.13	1.13	1.16
**Work status**	1.33	1.4	1.4	1.42
**Living arrangement**	1.85	1.87	1.92	1.92
**Monthly household income**	2.40	2.45	2.62	2.63
**Self-rated health**		1.24	1.25	1.29
**ADL**		1.67	1.68	1.69
**IADL**		1.78	1.78	1.82
Functional Assessment of Currently Employed Technology Scale			2.03	2.23
**Technostress**				1.20
**Technology use Self-efficacy**				2.16
**Technology Enthusiasm**				1.91

Note. All VIF values were below 5.0, indicating no serious multicollinearity.
